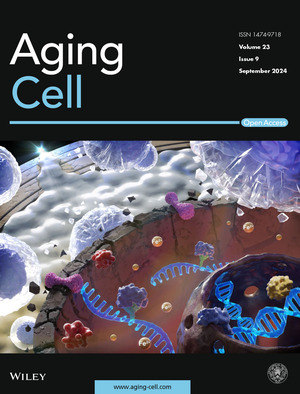# Featured Cover

**DOI:** 10.1111/acel.14346

**Published:** 2024-09-12

**Authors:** Xiao Lu, Dachuan Li, Zhidi Lin, Tian Gao, Zhaoyang Gong, Yuxuan Zhang, Hongli Wang, Xinlei Xia, Feizhou Lu, Jian Song, Guangyu Xu, Jianyuan Jiang, Xiaosheng Ma, Fei Zou

## Abstract

Cover legend: The cover image is based on the Article *HIF‐1α‐induced expression of the m6A reader YTHDF1 inhibits the ferroptosis of nucleus pulposus cells by promoting SLC7A11 translation* by Xiao Lu et al., https://doi.org/10.1111/acel.14210